# Gender Disparities in the Prevalence of Undernutrition and the Higher Risk among the Young Women of Indian Tribes

**DOI:** 10.1371/journal.pone.0158308

**Published:** 2016-07-05

**Authors:** Gautam K. Kshatriya, Subhendu K. Acharya

**Affiliations:** Department of Anthropology, University of Delhi, Delhi India; Queensland University of Technology, AUSTRALIA

## Abstract

**Background:**

High undernutrition is a grave concern in India. Marginalized populations like Indian tribes have been under the serious stress of such nutritional extreme. Women, in particular, are the worst sufferers. Gender-related comprehensive studies regarding the prevalence and risks of undernutrition among the tribes have not been properly pursued in India; the vulnerability of the young females has least been examined.

**Methods and Findings:**

We conducted a cross-sectional study during January 2011 to December 2013 among 1066 males and 1090 females (n = 2156) in the 20–60 years age group belonging to the nine major tribes; Santals, Oraons and Koras (West Bengal): Santals, Bhumijs and Bathudis (Odisha): Dhodias, Kuknas and Chaudharis (Gujarat). The undernutrition burden was estimated and such risks were analyzed for the women in comparison to the men. The overall undernutrition among the females was found to be 47.4% (95% CI 44.4–50.4) against 32.1% (95% CI 29.3–34.9) among males, indicating about a half of the female population undernourished. The odds of risks for underweight status among females were observed to be high in comparison to males with an odds of 1.9 (95% CI, 1.6–2.2; p≤0.001) for the overall undernutrition category, 1.7 (95% CI, 1.3–2.3; p≤0.001) for the mild undernutrition category, 1.3 (95% CI, 1.1–1.6; p≤0.01) for combined moderate and mild undernutrition category and 3.3 (95% CI at 2.3–4.6; p≤0.001) for severe undernutrition category. The young females were observed with a high prevalence of undernutrition along with increased risk. The 30-year mean BMI trend of the Indian population in comparison to the males, females, and overall tribal population places the tribal females at the highest risk.

**Conclusion:**

Indian tribes are suffering from the higher prevalence of undernutrition by further highlighting a high gender bias. The health and empowerment of adolescent and young tribal girls needs additional focus. Overall, no remarkable control on undernutrition has been achieved among Indian tribes despite various efforts.

## Introduction

Undernutrition is a consistent health problem among the children as well as the adult populations in India [1–4]. The prevalence is highest among the individuals from the marginalized sections of the Indian society like Scheduled Tribes (STs) [5] and Scheduled Castes (SCs) [6] than others [2]. Acute hunger is the single most contributing factor of undernutrition posing a serious concern for the country [7]. The Global Nutrition Report (2015) has also highlighted the high prevalence of undernutrition as a serious issue in India [4]. Furthermore, India ranked 135th out of 187 countries in the UNDP Human Development Index (2014) while Global Hunger Index (GHI), 2014 kept India in the ‘serious’ category by ranking it in the 55^th^ place among 79 countries; a status worse than many sub-Saharan countries and Asian countries like Bangladesh and Sri Lanka [8]. The latest research shows that 15.2% of the Indian population are undernourished [9]. Policy planners and social thinkers at national and international level have expressed their concern over the acute state of undernutrition in the country [10].

The challenges of widespread undernutrition among the infants and the children are the most important prevailing concerns in India [11, 12]. Government of India has been taking a focused and target-oriented approach to address this issue. Here, it needs emphasis that though adult undernutrition, particularly among the marginalized sections [Scheduled Tribes and Scheduled Castes] and vulnerable groups (women and aged) is also a serious health concern, a tangible policy in nutritional intervention program is yet to be prioritized in this direction. Individuals belonging to underprivileged sections and experiencing prolonged undernutrition during their early age, in most cases continue to suffer from undernutrition in the adulthood due to their persisting poor socioeconomic status and non-affordability towards modern health care [13, 14]. Lack of socioeconomic conduciveness among the individuals as well as the households from the marginalized sections has led to widespread undernourishment, where women, have suffered the worst health conditions. This has exacerbated the vicious circle of undernourishment and its effects which is auto-inherited to next generations in the family. Studies have shown that such children troubled by high undernutrition in their early age are at a higher risk of non-communicable diseases and other related complications in their adult age [15–18]. It is particularly true for the children belonging to the marginalized communities [19, 20]. It has further been witnessed among the non-tribal Indian populations that females are at an extreme odd with respect to childhood undernutrition in comparision to their male counterpart. A high prevalence of wasting (15%) and stunting (38.8%) among the children [4] with a substantial level of gender disparity (particularly in rural areas) [19, 21] is a leading cause of high undernutrition among the adult females. Moreover, it has been found that every second woman in India is anemic [19]. The prevalence rate of undernutrition among the ever married women stands at 55.3% as against 24.2% among the ever married men in India [22]. The huge gender gap shows high discrepancy against the women in accessing adequate food and diet. So, women’s vulnerability towards suffering from underweight in all ages is a serious concern in India. Nube (1998) inferred that undernutrition which is an indicator of poor health is also a significant indicator of the socioeconomic status of such individuals and households [23]. A high prevalence rate of 44.7% or more of undernutrition as well as anemia observed among the adolescent girls [11, 24] and a similar state of health among the young women [25] implies considerable discrimination against Indian women in the socioeconomic front. Factors like low social status, poverty, lack of education and marginalisation in accessing health and social care during early as well as later stages of life are the major causes for high nutritional discrepancies againest them [15]. Thus, such observations, highlighting the high-risk factors associated with undernutrition juxtapose the findings of various studies [19, 26] inferring no gender gap (in children aged <5 years and adolescents) in the prevalence of undernutrition among the tribes of India call for further thorough examination.

Tribal females constitute 4.2% (51 million) of the total Indian population [27]. It is a matter of grave concern that Indian tribes, and the tribal females in particular are vulnerable to the high prevalence and risk of nutritional extreme due to lack of adequate food and diet. There are few comprehensive and baseline studies measuring the under-nutrition status of the tribal populations. A large scale study on nutritional status of adults in Indian tribes was conducted by NNMB (National Nutrition Monitoring Bureau) in the year 2007–08 [26] which reported undernutrition to be prevalent among 49% of the women against 40% among the men. Similarly, the prevalence of undernutrition (<5 centile of BMI: thinness) among school age and adolescents was very high (6–9 years: 71%; 10–13 years: 61% & 14–17 years: 40%) [26].

Recent studies [28–32] on the nutritional status of the tribal women are few and such studies have reported a high prevalence and risk of undernutrition among females. In this context, the present study attempts to estimate the gender disparities in the prevalence of undernutrition among the nine major tribes of India, with a special emphasis on young women.

## Material and Methods

### Ethical statement

Prior ethical clearance from the Ethical Committee of Department of Anthropology, University of Delhi, Delhi was obtained to conduct the research. Informed written consent from the participants was obtained prior to the actual commencement of the study.

### Sample

**Fig 1:**
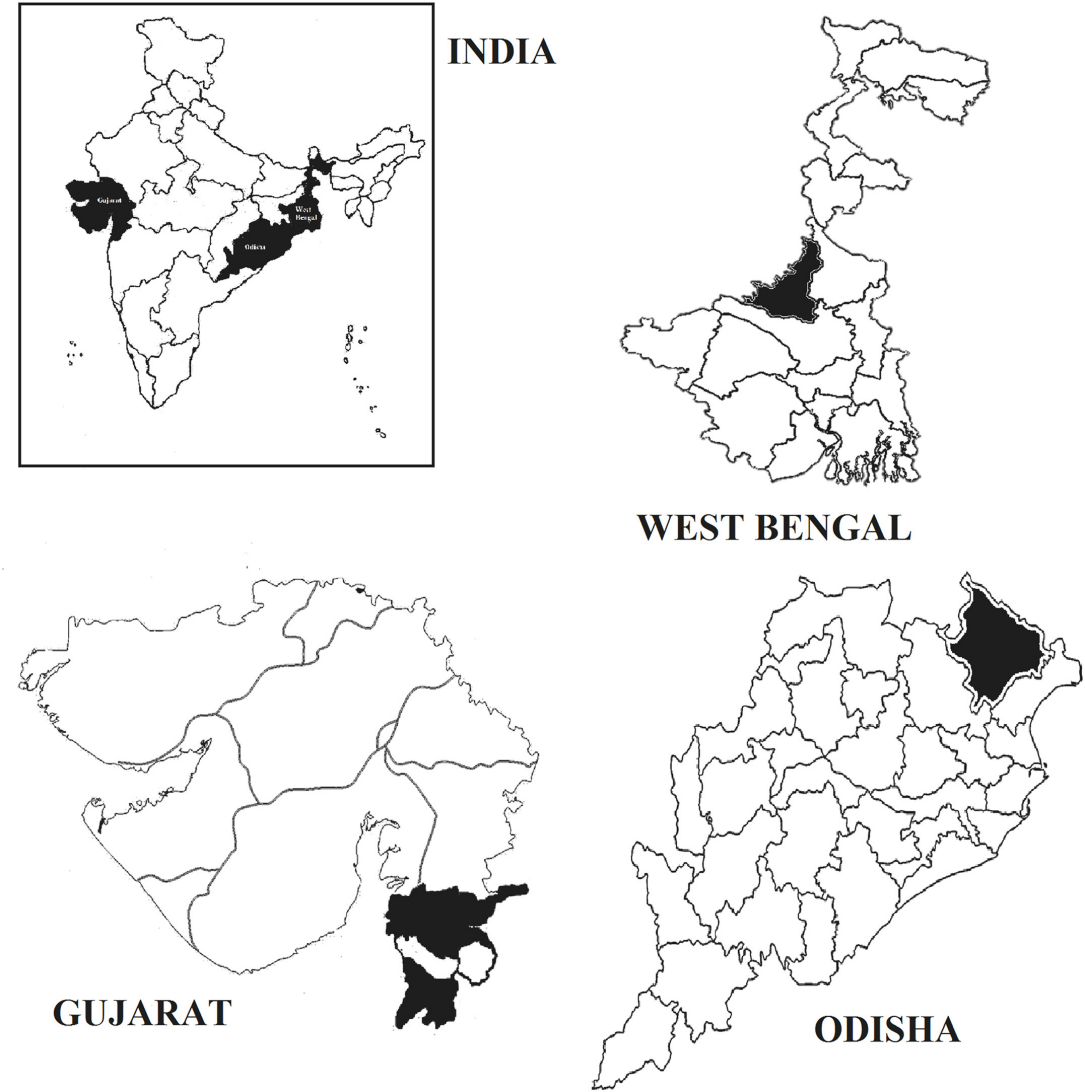
Geographic locations of the selected districts in West Bengal, Odisha, and Gujarat on the map of India.

The present study followed a multi-stage sampling method for sample collection. Three states were selected from two different regions of the country; two from the eastern region (West Bengal and Odisha) and one from the western region (Gujarat) (Fig 1). Three tribes, on the basis of their predominant distribution were selected from each state. Village listing for each of the tribes was prepared according to their population concentration. Furthermore, a total of 66 tribal villages from four districts were chosen in the three states on the basis of the residence in the acculturated areas of development (areas where developmental activities have reached). These areas have comparatively closer access to the ‘urban centers’.

House listing was prepared for these selected villages. Subsequently, the age and sex composition of four 10-year age interval groups (20–60 years) was obtained for the studied nine tribes. A sample size of 30 men and women from each of the four 10-year age interval groups was selected using systematic random sampling. A sample size of thirty participants was fixed for each age interval, though such targets could not be achieved by small margins in few of the age groups.

The study thus is comprised of a total sample size of 2156 adult tribal males and females belonging to the nine different tribes subdivided into four age cohorts of ten-year age intervals ranging from 20 years through 60 years. Criteria used for the exclusion were: growth and development disorders, severe health problems in last one year. The sample size of the present study was tested at 5% level of significance with a power of 80%. On average, 86% response rate was achieved.

### Field survey design

The present cross-sectional study was conducted between January 2011 and December 2013 in five different phases to collect data on the selected anthropometric measurements. Each tribe was identified as one cluster and data from every cluster was collected during a single period of time. All the participants were randomly selected.

### Composition of research team and sample collection method

Two trained anthropologists conducted the field research for the data collection. In order to avoid measurement and data entry bias, all the measurements were taken by one anthropologist while data and observations were recorded in the datasheet by the other. Participants who avoided the sampling were skipped.

### Measurement of Anthropometric Variables

Anthropometric measures have been most useful for evaluation of population-based nutritional status [33]. BMI (kg/m^2^) which is calculated based on two common physiological variables such as height and weight is one of the most useful and popular anthropometric measures over the world [33]. Most importantly, BMI eliminates the confounding effects of height on weight [34]. So in the present context, BMI was considered for understanding and explaining the status of undernutrition in the Indian tribal populations.

The primary information of the participants like their names, tribal identity, age and sex were recorded in a structured schedule before taking anthropometric measurements. Standard techniques were followed while taking all the anthropometric measurements [35]. Standing height and weight was measured to the nearest of 0.1 centimeters (cm) and 0.1 kilograms (kg) respectively. Stature was measured by using movable anthropometer; weight was measured by using an Omron Karada Scan Body Composition Monitor. The participants were encouraged to remove their shoes and heavy clothing before giving the measurements. BMI was calculated as weight in kilogram (kg) divided by height in meter squared (m^2^)—kg/m^2^.

### Individual classifications

BMI, the standard indicator of undernutrition in the present study was divided into various categories based on the Chronic Energy Deficiency (CED) grades given by Ferro-Luzzi et al. (1992); individuals with BMI<18.5 kg/m^2^ were considered as underweight; BMI ≥17 to <18.5 kg/m^2^ as moderately underweight or CED grade I; BMI of ≥16 to <17 kg/m^2^ were mild underweight or CED grade II; BMI of <16 kg/m^2^ as severely underweight or CED grade III [36]. A fifth category was formed by combining the mild and moderate categories together for the present analysis purpose.

### Statistical Analysis

After incorporating and systematising the data in Microsoft Excel 2007, analysis was carried out using Microsoft Excel 2007 and SPSS Version 16.0 for Windows, SPSS Inc., Chicago, Illinois, USA. In order to maintain the integrity of the sample data, double entry of the data was done. Multiple-time crosscheck of the data was conducted to maintain the validity of the data so that the positive, negative and decimal characters of the numbers in the data could be ensured. Sex wise descriptive statistics such as mean and standard deviation (SD) were estimated for the selected anthropometric variable (BMI) for each of the tribes as well as on the whole. The prevalence of selected risk categories of undernutrition in males, females as well as in the overall population were estimated. Subsequently, 95% CI was constructed for each prevalence rate in the young and older population categories by using Wilson model without continuity correction [37]. The estimated mean BMI of each tribe among the males and the females as well for the total population was presented in bar graphs. Odds for females against males in each category of undernutrition were estimated in multivariate method by using logistic regression model. Similarly, age-related odds ratios of undernutrition across the four selected categories were also calculated by using the same above method for <40 years and ≥40 years of females in comparison to their male counterparts. P value falling between 0.09 and 0.05 was considered as suggestive which has been represented as 0.09≥p≥0.05 [38].

## Results

Table 1 describes the sample size for both the males and the females for each tribe. The mean BMI (±SD) for males and females in each tribe demonstrates a profound variation. It highlights the gender gap in food security among tribal populations. Though the condition is clearly pronounced among females of all the tribes, the mean BMI among Kora, Oraon and Bathudi females, in particular, were observed to be lower than the normal BMI range-18.5–22.99 kg/m^2^. A high prevalence status of undernutrition though cannot be defined only on the basis of mean BMI, a mean BMI status in below normal level among women than the men clearly indicates a high gender disparity in the nutritional status of the studied groups. Additionally, an observed low mean BMI in a population signifies the prevalence of either acute malnutrition or undernutrition among a sizable section of the population. These aspects have further been analyzed in the following sections.

**Table 1:** Demographics and population characteristics of the selected tribes.

Name of the tribes	Gender	Sample Size	BMI (Mean±SD)
**Santal (WB)**	Males	123	19.9±2.6
	Females	122	19.5±3.2
	Total	245	19.7±2.9
**Kora**	Males	114	18.9±2.0
	Females	121	17.6±2.9
	Total	235	18.3±2.6
**Oraon**	Males	112	19.6±2.5
	Females	124	18.1±2.7
	Total	236	18.8±2.8
**Santal (O)**	Males	121	20.2±2.8
	Females	119	20.3±3.0
	Total	240	20.3±2.9
**Bhumij**	Males	116	20.9±3.1
	Females	122	19.7±2.9
	Total	238	20.3±3.1
**Bathudi**	Males	119	19.5±2.7
	Females	121	18.0±2.8
	Total	240	18.6±3.3
**Dhodia**	Males	121	20.5±3.2
	Females	120	20.7±3.5
	Total	240	20.6±3.3
**Kukna**	Males	120	20.3±3.1
	Females	120	19.9±2.9
	Total	240	20.1±3.0
**Chaudhari**	Males	120	19.8±3.1
	Females	121	18.9±2.9
	Total	241	19.4±3.0
**Total**	Males	1066	20.0±2.9
	Females	1090	19.2±3.1
	Total	2156	19.6±3.1

**Fig 2:**
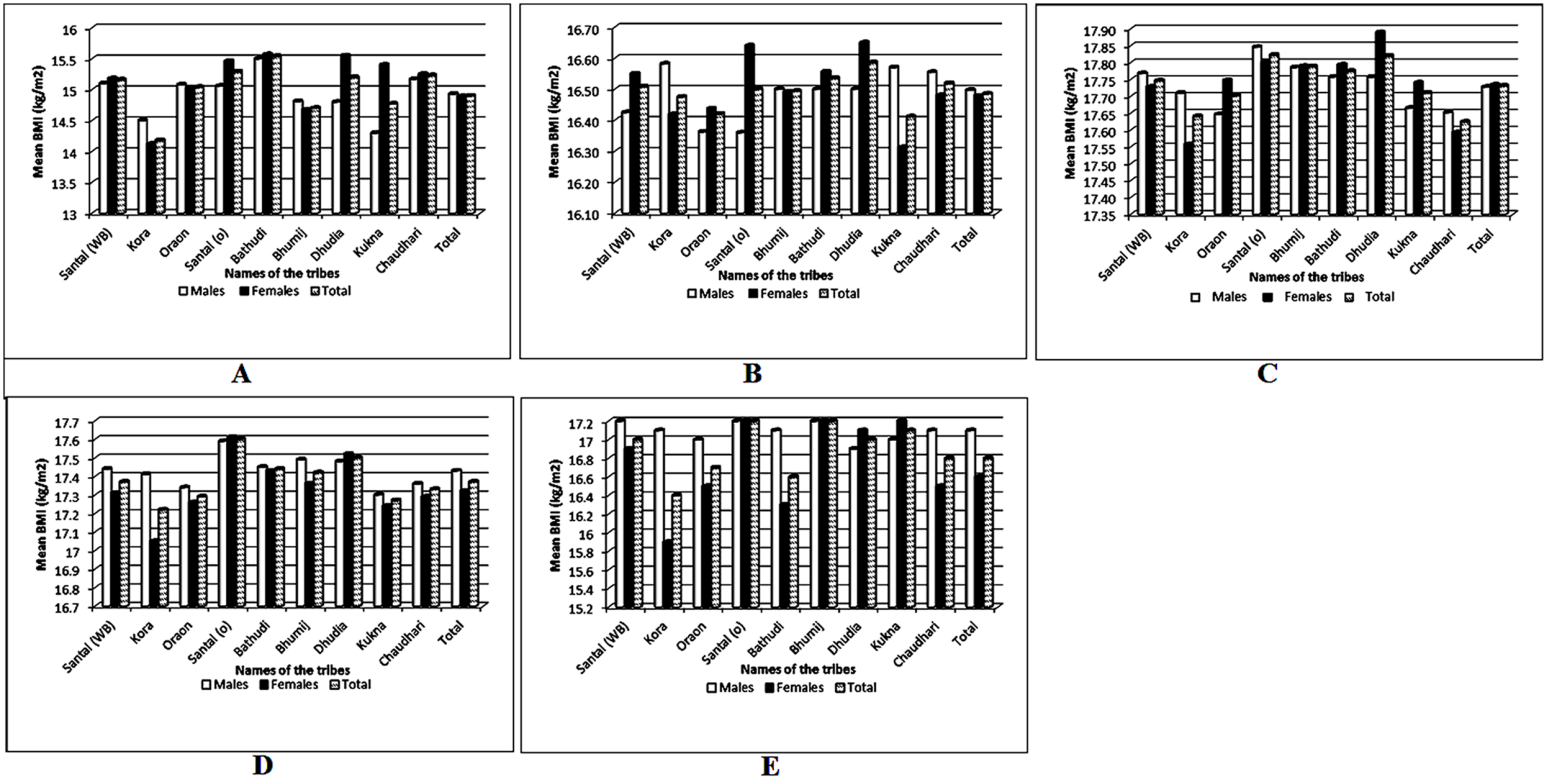
(A-E): Distribution of mean BMI in selected undernutrition categories among the nine tribes; A-severe undernutrition; B-moderate undernutrition; C-mild undernutrition; D-combined moderate and mild undernutrition; E-overall undernutrition.

This study attempted to obtain the actual gaps in BMI status of the females in comparison to the males. We also tried to examine the status of undernutrition in the studied tribes. The mean BMI among males and females was calculated in five selected categories; overall undernutrition, moderate undernutrition (Grade-I), mild undernutrition (Grade-II), severe undernutrition (Grade-III) and combined mild and moderate undernutrition. The findings were plotted in the form of bar graphs for five selected undernutrition categories; overall undernutrition along with mild, moderate, combined mild and moderate and severe forms (Fig 2A–2E). The comparison among these figures shows that there is a huge gender gap at the level of overall undernutrition (Fig 2E) except for Dhodia and Kukna from Gujarat and Bhumij from Odisha. Most importantly, in severe undernutrition condition (Fig 2A) the mean BMI among females was comparatively higher than the males among several of the selected tribes. On the other hand, Fig 2B shows that the prevalence of moderate undernutrition is very high among the female members of the tribes across the three states.

(Table 2A and 2B) represents the tribe wise status of undernutrition prevalence among the females in comparison to their male counterparts.

**Table 2:** (A-B): Prevalence rate (with 95% CI) of undernutrition in its various categories among tribal males and females.

**A**											
Sl No	**Name of the tribe**	**Prevalence of severe undernutrition (%; 95% CI)**				**Prevalence of moderate undernutrition (%; 95% CI)**			**Prevalence of mild undernutrition (%; 95% CI)**		
		**Males**	**Females**	**Total**		**Males**	**Females**	**Total**	**Males**	**Females**	**Total**
1	**Santal (WB)**	3.3 (1.1–8.6)	9.8 (5.4–16.9)	6.5 (3.9–10.6)		6.5 (3.1–12.8)	13.1 (7.9–20.7)	9.8 (6.5–14.4)	20.3 (13.8–28.7)	23.8 (16.7–32.5)	22.0 (17.1–27.9)
2	**Kora**	4.4 (1.6–10.4)	25.6 (18.3–34.5)	15.3 (11.1–20.7)		9.7 (5.2–17.0)	17.4 (11.3–25.5)	13.6 (9.6–18.8)	27.2 (19.5–36.5)	21.5 (14.8–30.1)	24.3 (19.0–30.4
3	**Oraon**	5.4 (2.2–11.8)	22.6 (15.8–31.1)	14.4 (10.3–19.7)		7.1 (3.4–14.0)	15.3 (9.7–23.2)	11.4 (7.8–16.4)	23.2 (16.0–32.3)	25.8 (18.6–34.6)	24.6 (19.3–30.7)
4	**Santal (O)**	4.1 (1.5–9.9)	5.9 (2.6–12.2)	5.0 (2.8–8.8)		4.1 (15.3–9.9)	4.2 (1.6–10.0)	4.2 (2.1–7.8)	19.8 (13.4–28.3)	21.0 (14.3–29.6)	20.4 (15.6–26.2)
5	**Bhumij**	3.5 (1.1–9.1)	4.9 (2.0–10.9)	4.2 (2.2–7.8)		4.3 (1.6–10.5)	9.0 (4.8–15.9)	6.7 (4.0–10.9)	12.1 (7.0–19.8)	23.8 (16.7–32.5)	18.1 (13.5–23.7)
6	**Bathudi**	5.9 (2.6–12.2	24.8 (17.6–33.6)	15.4 (11.2–20.8)		7.6 (3.7–14.3	13.2 (8.0–20.9)	10.4 (7.0–15.2)	27.7 (20.1–36.8)	24.8 (17.6–33.6)	26.3 (20.9–32.4)
7	**Dhodia**	5.8 (2.6–12.0)	6.7 (3.1–13.1)	6.2 (3.7–10.3)		5.0 (2.0–10.9)	6.7 (3.1–13.1)	5.8 (3.3–9.8)	17.4 (11.3–25.5)	15.8 (10.0–23.9)	16.6 (12.3–22.0)
8	**Kukna**	3.5 (1.1–9.3)	2.5 (0.7–7.7)	2.9 (1.3–6.2)		8.3 (4.3–15.2)	13.3 (8.1–21.0)	10.8 (7.3–15.6)	16.7 (10.7–24.8)	25.0 (17.8–33.9)	20.8 (16.0–26.6)
9	**Chaudhari**	5.8 (2.6–12.1)	19.0 (12.7–27.4)	12.5 (8.7–17.5)		9.2 (4.9–16.2	8.3 (4.3–15.0)	8.7 (5.6–13.2)	25.8 (18.5–34.8)	22.3 (15.5–31.0	24.1 (18.9–30.1)
10.	**Total**	4.6 (3.5–6.1)	13.6 (11.6–15.8)	9.1 (8.0–10.5)		7.2 (5.8–9.0)	11.1 (9.3–13.2)	9.2 (8.0–10.5)	21.1 (18.7–23.7)	22.7 (20.2–25.3	21.9 (20.2–23.8)
**B**											
**Sl No**	**Name of the tribe**	**Prevalence of mild and moderate (combined) undernutrition (%; 95% CI)**				**Overall prevalence of undernutrition (%; 95% CI)**					
		**Males**	**Females**		**Total**	**Males**	**Females**	**Total**			
1	**Santal (WB)**	26.8 (19.4–35.7)	36.9 (28.5–46.2)		31.8 (26.1–38.1)	30.1 (22.0–38.2)	45.1 (36.3–53.9)	37.6 (31.5–43.6)			
2	**Kora**	36.8 (28.2–46.4)	38.8 (30.2–48.2)		37.9 (31.7–44.4)	41.2 (32.2–50.3)	62.0 (53.3–70.6)	51.9 (45.5–58.3)			
3	**Oraon**	30.4 (22.2–39.99)	41.1 32.5–50.3)		36.0 30.0–42.5)	34.8 (26.0–43.6)	62.9 (54.4–71.4)	49.6 (43.2–56.0)			
4	**Santal (O)**	24.0 (16.9–32.7)	25.2 (17.9–34.2)		24.6 (19.4–30.6)	28.1 (20.1–36.1)	31.1 (22.8–39.4)	29.6 (23.8–35.4)			
5	**Bhumij**	16.4 (10.4–24.7)	32.8 (24.7–42.0)		24.8 (19.5–30.9)	19.0 (11.8–26.1)	36.9 (28.3–45.5)	28.2 (22.4–33.9)			
6	**Bathudi**	35.3 (26.9–44.6)	38.0 (29.5–47.3)		36.7 (30.6–43.1)	39.5 (30.7–48.3)	62.8 (54.2–71.4)	51.3 (44.9–57.6)			
7	**Dhodia**	22.5 (15.6–31.2)	22.5 (15.6–31.2)		22.5 (17.5–28.4)	28.1 (20.1–36.1)	29.2 (21.0–37.3)	28.8 (23.0–34.5)			
8	**Kukna**	25.4 (18.2–34.4)	38.3 (29.7–47.7)		31.9 (26.1–38.3)	28.3 (25.5–42.5)	40.0 (31.2–48.8)	34.2 (28.2–40.2)			
9	**Chaudhari**	35.0 (26.7–44.3)	30.6 (22.7–39.7)		32.8 (27.0–39.2)	40.0 (31.2–48.8)	48.8 (39.9–57.7)	44.4 (38.1–50.7)			
10	**Total**	28.0 (25.3–30.8)	33.9 (31.1–36.8)		30.9 (29.0–33.0)	32.1 (29.3–34.9)	47.4 (44.4–50.4)	39.4 (37.4–41.5)			

Table 2A and 2B show that tribal females are the worst sufferers of the nutritional health hazard in all the five undernutrition categories.

The prevalence of severe undernutrition among the women is 13.6% (95% CI, 11.6–15.8) against 4.6% (95% CI, 3.5–6.1) among the males; there is a 9.1% (95% CI, 8.0–10.6) prevalence of overall severe undernutrition (Table 2A). The male-female gap in the severe category of undernutrition is huge with the females in 5 of the 9 tribes, showing a higher prevalence of undernutrition than their male counterparts (Table 2A).

Gender disparity in the prevalence of moderate undernutrition was observed in 6 out of 9 studied tribes. Overall, the prevalence rate of moderate undernutrition is 11.1% (9.3–13.2) among the females as compared to 7.2% (5.8–9.0) among the males (Table 2A). In the mild undernutrition category, the differences in the prevalence of undernutrition among the males and the females are less pronounced (Table 2A). The prevalence of combined mild and moderate undernutrition was found to be 33.9% (95% CI, 31.1–36.8) among the women as against 28 percent among the men (95% CI, 25.3–30.8); women in 7 of the 9 tribes show higher prevalence than the men (Table 2B).

The overall undernutrition (Table 2B) among the tribal females was observed to be 47.4% (95% CI, 44.4–50.4) as against 32.1% for the tribal males (95% CI, 29.3–34.9); the females of all the nine tribes showed higher prevalence in this category than the males. Tribes like Kora, Oraon and Bathudi tribes need special mention as the women of these tribes show a 60% of prevalence in the overall undernutrition category (BMI<18.5 kg/m^2^) as compared to the men who show less than 42% prevalence of overall undernutrition.

Some differences from the above trend of high gender difference were also observed. Dhodias of Gujarat and Santals of Odisha are two such tribes that exhibit a picture of equal nutritional status among the males and the females. Further, in the mild and moderate undernutrition category, Chaudhari males show a higher prevalence than Chaudhari females.

**Fig 3:**
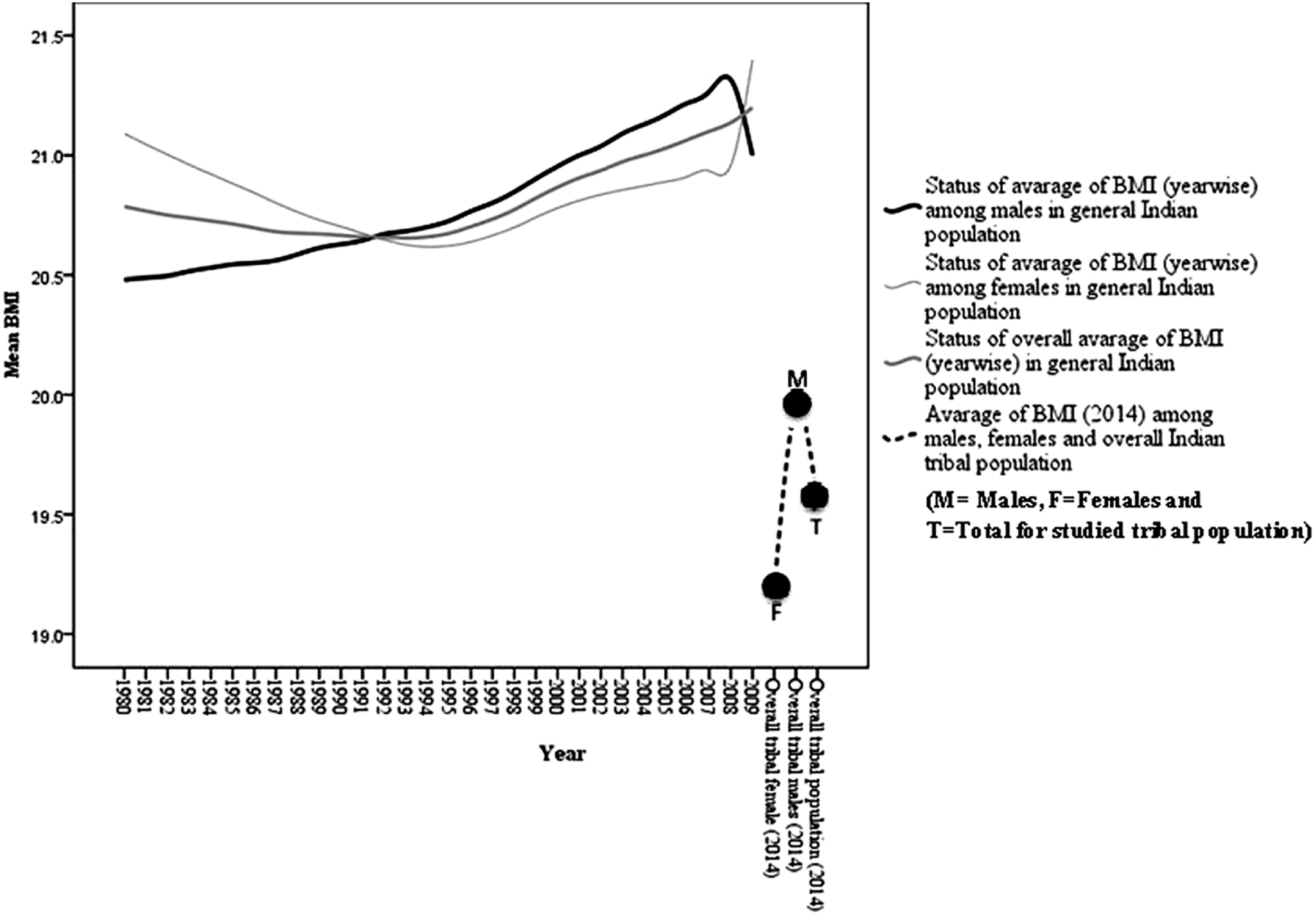
Thirty year trend of BMI in general population of India along with the observed BMI pattern among the pooled tribal populations in the present study.

In the absence of comprehensive data in literature on BMI trend of Indian tribes in past years, the present paper has provided a detailed analysis regarding BMI in nine major tribes of India. We analyzed the last 30 year trend of BMI in the males and the females of the Indian population [39] and compared it with the present findings (Fig 3), which showed a dismal picture of the tribal women.

Since the prevalence of undernutrition in tribal women was high, we compared it with its corresponding prevalence in tribal men (Table 3). The analysis produced important findings with highly significant risks among the females in all the five categories of undernutrition. In the severe undernutrition category in particular, the females were observed to be at more than 3 times higher risk than the males to suffer from such nutritional adversity. While calculating the odds of being underweight, females were observed to be at highly significant risk than males; for the overall undernutrition, odds ratio is 1.9 (95% CI, 1.6–2.2; p≤0.001); for mild undernutrition, odds ratio is 1.7 (95% CI, 1.3–2.3; p≤0.001); for combined moderate and mild undernutrition, odds ratio is 1.3 (95% CI at 1.1–1.6; p≤0.01); for severe undernutrition, odds ratio is 3.3 (95% CI, 2.3–4.6;p≤0.001). These observations explain that the prevalence of undernutrition among females has considerably risen in comparison to males with a steep increase in the severity categories. Most importantly, Dhodias of Gujarat showed a very encouraging picture of nutrition among the males and the females.

**Table 3:** The odds for females against male undernutrition in selected categories among the tribes.

Sl No	Name of the tribe	Odds for overall undernutrition (<18.5 kg/m2)	Odds for moderate undernutrition (≥16 to <16.99 kg/m2)	Odds for mild undernutrition (≥17 to <18.5 kg/m2)	Odds for moderate+mild undernutrition (≥16 to <18.5 kg/m2)	Odds for severe undernutrition (<16 kg/m2)
1	Santal (WB)	1.9 (1.1–3.2)*	2.2 (0.9–5.3)#*	1.2 (0.7–2.2)	1.6 (0.9–2.7)#*	3.3 (1.0–10.36)*
2	Kora	2.3 (1.4–3.9)**	2.0 (0.9–4.3)#*	0.7 (0.4–1.3)	1.1 (0.6–1.9)	7.5 (2.8–20.1)***
3	Oraon	3.2 (1.9–5.4)***	2.4 (1.0–5.6)#*	1.2 (0.6–2.1)	1.6 (0.9–2.8)#*	5.2(2.1–13.0)**
4	Santa (O)	1.2 (0.7–2.0)*	1.0 (0.3–3.6)	1.1 (0.6–2.0)	1.1 (0.6–1.9)	1.5 (0.5–4.7)
5	Bhumij	**2.5 (1.4–4.5)** **	2.2 (0.7–6.5)	2.3 (1.1–4.6)*	2.5 (1.3–4.6)**	1.5 (0.4–5.3)
6	Bathudi	2.6 (1.5–4.4)***	1.9 (0.8–4.4)	0.89 (0.5–1.5)	1.1 (0.7–1.9)	5.3 (2.2–12.6)***
7	Dhodia	1.1 (0.6–1.8	1.4 (0.5–4.1)	0.9 (0.5–1.8)	1.01 (0.6–1.9)	1.2 (0.4–3.3)
8	Kukna	1.7 (1.0–2.9) #*	1.7 (0.7–3.9)	1.7 (0.9–3.1)	1.9 (1.2–3.2)*	0.7 (0.2–3.4)
9	Chaudhari	1.4 (0.9–2.4)	0.9 (0.4–2.2)	0.8 (0.5–1.5)	0.8 (0.5–1.4)	3.8 (1.6–9.2)**
10	Total	1.9 (1.6–2.2)***	1.7 (1.3–2.3)***	1.1 (0.9–1.3)	1.3 (1.1–1.6)**	3.3 (2.3–4.6)***

*p is significant at ≤0.05,

** p is significant at ≤0.01,

*** p is significant at ≤0.001,

^#^*p is 0.09≥p≥0.05.

Observing the high risk of undernutrition among the women, we further examined such disparities in the prevalence of risk in different age groups. So, an analysis on prevalence of undernutrition (Tables 4–6) was undertaken among the females in the young and the older age groups by comparing them to their male counterparts in the corresponding age groups. This analysis was conducted in the same five categories of undernutrition. It was observed that females in the age group of less than 40 years of age and in all the five categories of undernutrition show higher prevalence than males in the corresponding age groups (Tables 4–6) with a fewer exceptions; Dhodias and Santals (WB) in the severe category, Chaudharis and Koras in mild undernutrition category and Chaudharis in combined moderate and mild undernutrition category.

**Table 4:** Prevalence of undernutrition in its various categories in <40 year and ≥40 year age groups among males and females in the Santal (O), Bathudi and Bhumij tribes.

Prevalence type		Santal (O)		Bathudi		Bhumij	
	Sex	<40 years	≥40 years	<40 years	≥40 years	<40 years	≥40 years
**Undernutrition<18.5 kg/m**^**2**^	M	20.3 (11.4–33.2)	34.4 (23.0–47.8)	35.5 (24.0–48.7)	47.4 (34.2–60.9)	7.3(2.4–18.4)	31.2(20.25–44.4)
	F	42.4 (29.9–55.9)	20.0 (11.2–32.7)	63.9 (50.6–75.5)	61.7 (48.2–73.7)	30.7 (19.9–43.8)	45.0 (32.3-58-3)
**Mild undernutrition≥17 to <18.5 kg/m**^**2**^	M	15.3 (7.6–27.5)	23.0 (13.5–35.8)	7.3 (2.4–18.4)	16.4 (8.6–28.5)	25.8 (15.9–38.7)	29.8 (18.8–43.6)
	F	30.5 (19.5–44.0)	11.7 (5.2–23.2)	21.8 (12.3–35.4)	28.3 (17.8–41.6)	29.5 (18.9–42.7)	20.0 (11.2–32.7)
**Moderate undernutrition ≥16 to <16.99kg/m**^**2**^	M	3.4(0.6–12.8)	4.9 (1.3–14.6)	0.0	8.2 (3.1–18.8)	6.5 (2.1–16.5)	8.8 (3.3–20.0)
	F	6.8 (2.2–17.3)	1.7 (0.1–10.1)	6.5 (2.1–16.5)	11.7 (5.2–23.2)	13.1 (6.2–24.8)	13.3 (6.3–25.1)
**Combined moderate and mild undernutrition≥16 to <18.5 kg/m**^**2**^	M	18.6 (10.1–31.3)	28.3 (17.8–41.6)	32.3 (21.3–45.5	38.6 (26.3–52.4)	7.3 (2.4–18.4)	24.6 (14.9–37.6)
	F	37.3 (25.3–50.9)	13.3 (6.3–25.1)	42.6 (30.3–55.9)	33.3 (22.0–46.8)	25.8 (15.9–38.7)	40.0 (27.8–53.5)
**Severe ndernutrition <16 kg/m**^**2**^	M	1.7 (0.1–10.3)	6.7 (2.2–17.0)	3.2 (0.6–12.2)	8.8 (3.3–20.0)	0.0	6.6 (2.1–16.8)
	F	5.1 (1.3–15.1)	6.7 (2.2–17.0)	21.3 (12.3–34.0)	28.3 (17.8–41.6)	4.8 (1.3–14.4)	5.0 (1.3–14.8)

**Table 5:** Prevalence of undernutrition in its various categories in <40 year and ≥40 year age groups among males and females in the Santal (WB), Kora and Oraon tribes.

Prevalence type		Santal (WB)		Kora		Oraon	
	Sex	<40 years	≥40 years	<40 years	≥40 years	<40 years	≥40 years
**Undernutrition <18.5 kg/m**^**2**^	M	28.6 (18.2–41.5)	31.7 (20.6–45.1)	39.0 (26.8–52.6)	43.6 (30.6–57.6)	25.4 (15.4–38.7)	47.2 (33.5–61.2)
	F	50.0 (37.2–62.8)	43.3 (30.8–56.7)	59.0 (45.7–71.2)	70.0 (56.6–80.8)	69.8 (56.8–80.4)	57.4 (44.1–69.7)
**Mild undernutrition ≥17 to <18.5 kg/m**^**2**^	M	20.6 (11.9–33.0)	20.0 (11.2–32.7)	25.4 (15.4–38.7)	29.1(18.0–43.1)	22.0 (12.7–35.1)	24.5 (14.2–38.6)
	F	25.8 (15.9–38.7)	21.7 (12.5–34.5)	23.0 (13.5–35.8)	20.0 (11.2–32.7)	36.5) 25.0–49.7)	14.8 (7.4–26.7)
**Moderate undernutrition ≥16 to <16.99 kg/m**^**2**^	M	4.8 (1.2–14.2)	8.3 (3.1–19.1)	10.2 (4.2–21.5)	9.1 (3.4–20.7)	3.4 (0.6–12.8)	11.3 (4.7–23.7)
	F	12.9 (6.1–24.4)	13.3 (6.3–25.1)	18.2 (9.5–31.4)	18.3 (9.9–30.9)	24.5 (14.2–38.6)	9.8 (4.1–20.9)
**Combined moderate and mild undernutrition ≥16 to <18.5 kg/m**^**2**^	M	25.4 (15.6–38.2)	28.3 (17.8–41.6)	35.6 (23.9–49.2)	38.2 (25.7–52.3)	25.4 (15.4–38.7)	35.9 (23.5–50.3)
	F	38.7 (26.9–52.0)	35.0 (23.5–48.5)	39.3 (27.3–52.7)	38.3 (26.4–51.8)	57.1 (44.1–69.3)	24.6 (14.9–37.6)
**Severe undernutrition <16 kg/m**^**2**^	M	3.2 (0.6–12.0)	3.34 (0.58–12.54)	3.4 (00.6–12.8)	5.5 (1.4–16.1)	0.0	11.3 (4.7–23.7)
	F	11.3 (5.0–22.5)	8.3 (3.1–19.1)	19.7 (11.0–32.2)	31.7 (20.6–45.1)	12.7 (6.0–24.1)	32.8(21.6–46.1)

**Table 6:** Prevalence of undernutrition in its various categories in <40 year and ≥40 year age groups among males and females in the Dhodia, Kukna and Chaudhari tribes along with the total pooled tribal population.

Prevalence type		Dhodia		Kukna		Chaudhari		Overall	
	Sex	<40 years	≥40 years	<40 years	≥40 years	<40 years	≥40 years	<40 years	≥40 years
**Undernutrition <18.5kg/m**^**2**^	M	29.5 (18.9–42.7)	27.1 (16.8–40.5)	22.6 (13.3–35.3)	34.5 (22.8–48.2)	38.3 (26.4–51.8)	43.3 (30.8–56.7)	27.5 (23.9–31.6)	37.6 (33.5–41.9)
	F	40.0 (27.8–53.5)	18.3 (9.9–30.9)	43.3 (30.8–56.7)	38.3 (26.4–51.8)	45.0 (32.3–58.3)	54.1 (40.9–66.7)	49.5 (45.2–53.7)	45.4 (41.2–49.7)
**Mild undernutrition≥17 to <18.5 kg/m**^**2**^	M	19.4 (10.8–31.7)	15.3 (7.6–27.5)	14.5 (7.3–26.3)	19.0 (10.3–31.8)	26.7 (16.5–39.9)	25.0 (15.1–38.1)	19.8 (16.6–23.4)	22.3 (18.9–26.2)
	F	21.7 (12.5–34.5)	10.0 (4.1–21.2)	26.7 (16.5–39.9)	23.3 (13.8–36.3)	18.3 (9.9–30.9)	26.2 (16.2–39.3)	25.7 (22.2–29.6)	19.56 (16.4–23.2)
**Moderate undernutrition≥16 to <16.99 kg/m**^**2**^	M	1.6 (0.1–9.8)	8.5 (3.2–19.4)	4.8 (1.3–14.4)	12.1 (5.4–23.9)	6.7 (2.2–17.0)	11.7 (5.2–23.2)	4.6 (3.1–6.8)	9.2 (6.9–12.0)
	F	11.7 (5.2–23.2)	1.7 (0.1–10.1)	11.7 (5.2–23.2)	15.0 (7.5–27.1)	6.7 (2.2–17.0)	9.8 (4.1–20.9)	11.9(9.3–14.9)	10.5 (8.1–13.5)
**Combined moderate and mild undernutrition≥16 to <18.5 kg/m**^**2**^	M	21.3 (12.3–34.0)	23.7 (14.0–36.9)	19.4 (10.8–31.7)	31.0 (19.9–44.7)	33.3 (22.0–46.8)	36.7 (24.9–50.2)	24.4 (20.9–28.3	31.5 (27.6–35.7)
	F	33.3 (22.0–46.8)	11.7 (5.2–23.2)	38.3 (26.4–51.8)	38.3 (26.4–51.8)	25.0 (15.1–38.1)	36.1 (24.5–49.4)	37.6 (33.6–41.8)	30.1 (26.3–34.2)
**Severe undernutrition<16 kg/m**^**2**^	M	8.2 (3.1–18.8)	3.39 (0.59–12.75)	3.2 (0.6–12.2)	3.5 (0.6–13.0)	5.0(1.3–14.8)	6.7 (2.2–17.0)	3.1 (1.9–5.1)	6.1 (4.3–8.6)
	F	6.7 (2.2–17.0)	6.7 (2.2–17.0)	5.0 (1.3–14.8)	0.0	20.0 (11.2–32.7)	18.0 (9.8–30.4)	11.9(9.3–14.9)	15.3 (12.4–18.7)

Furthermore, while clubbing all the tribes together young tribal females show higher prevalence in all of the 5 categories of undernutrition as compared to the males (Table 6).

Similarly, young tribal women show a higher prevalence of undernutrition in the four of the five categories (except for severe undernutrition) as compared to the older women (>40 years) (Table 6).

The present findings also indicate a huge gender gap in the prevalence of undernutrition in its various categories in each tribe as well as all the tribes pooled together while sub-divided by age groups (Tables 4–6).

In the older age group, while the prevalence rate among the females in the overall (45.4%, 95% CI 41.2–49.7) and severe (15.3%, 95% CI 12.4–18.7) undernutrition categories is higher in comparison to the males (with 37.6%, 95% CI 33.5–41.9 and 6.1%, 95% CI 4.3–8.6) in the respective categories, it was observed that the prevalence rate of combined mild and moderate undernutrition was higher (though marginally) among the older males (31.5%, 95% CI 27.6–35.7) than the females (30.1%, 95% CI 26.3–34.2).

**Fig 4:**
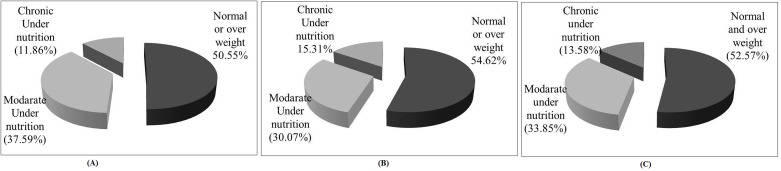
Prevalence of undernutrition among the tribal females in <40 years (A), ≥40 years (B) and the overall (C) categories.

Fig 4 represents the distribution pattern of undernutrition prevalence among the females in the <40 years (A) and ≥40 years (B) age group categories along with the total pooled population (C). It shows about half of the female population in each of the three categories are suffering from undernutrition.

**Fig 5:**
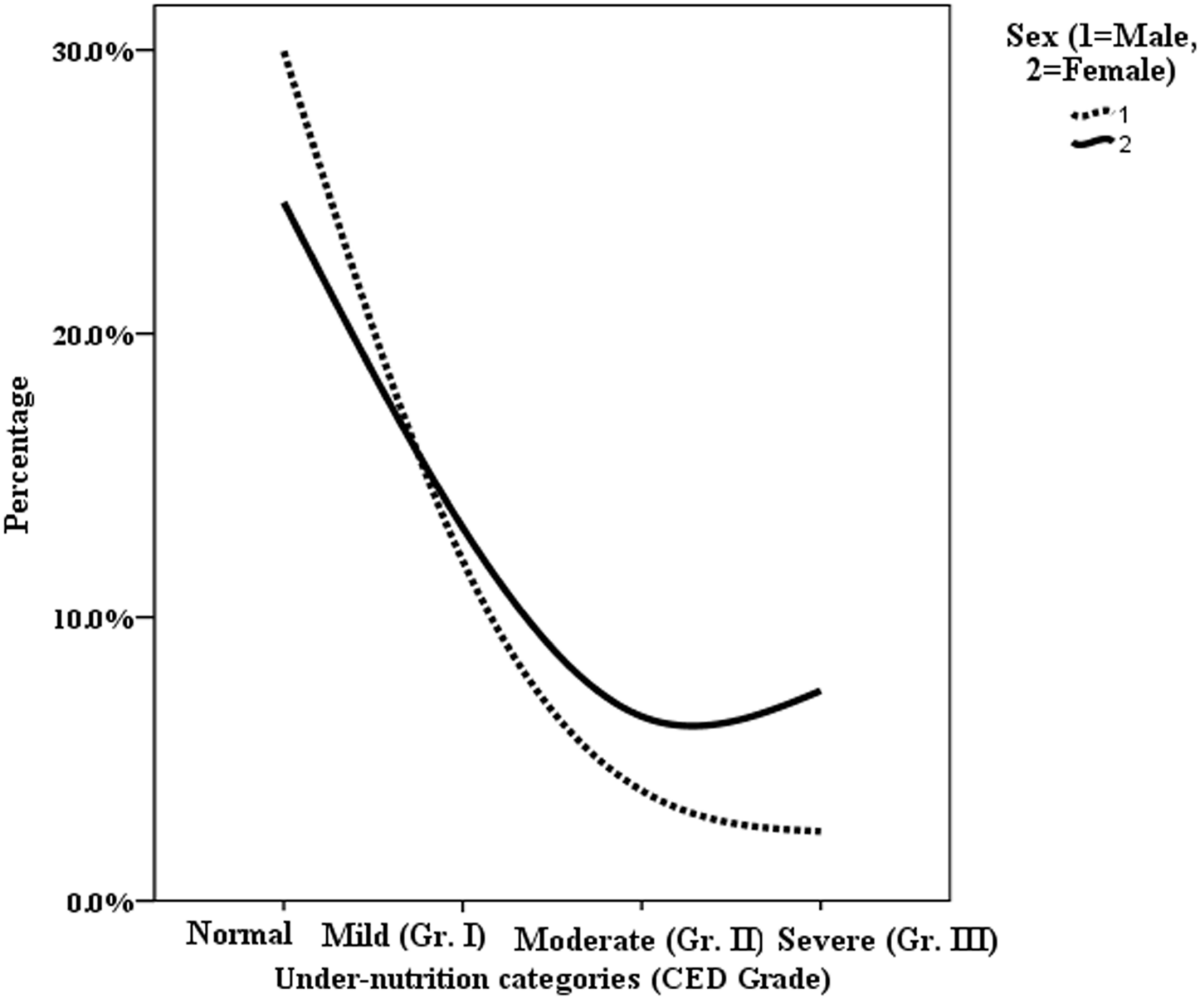
Sex wise grades of CED among Indian tribal population.

**Fig 6:**
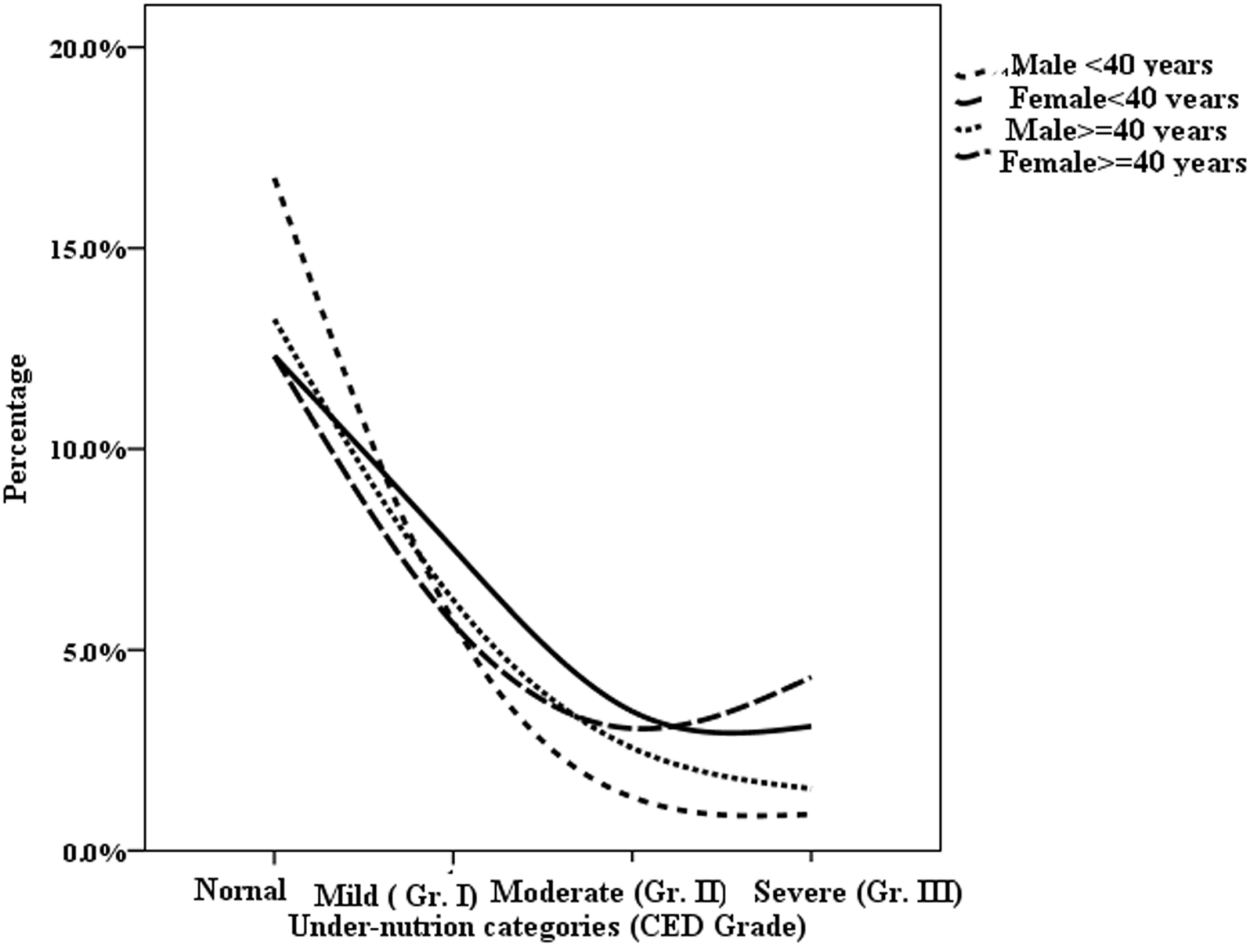
Age wise grades of CED among Indian tribal population.

Figs 5 and 6 represent the age and sex wise differential status of undernutrition respectively in terms of CED (chronic energy deficiency) among the selected participants which highlight a high gender gap in attending food security.

**Fig 7:**
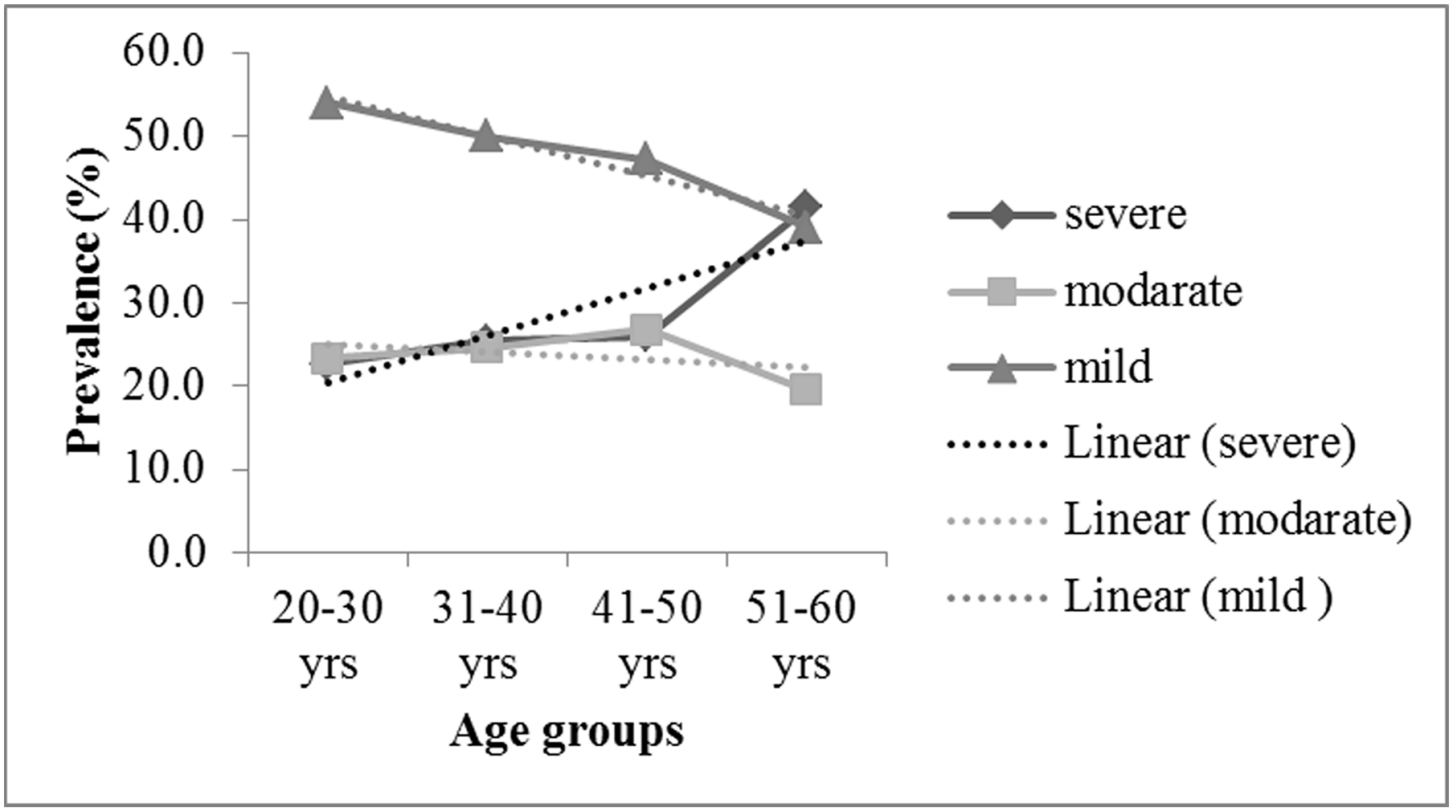
Prevalence and trend of undernutrition among four age categories.

We further plotted the individuals in 20–60 years age group by dividing in to four age cohorts (20–30 years, 31–40 years, 41–50 years and 51–60 years) for a more detailed analysis of on the undernutrition prevalence (Fig 7).

Table 7A represents the results of estimated risks among the females in comparison to the males in young (<40 years) and older (≥40 years) age groups in the five undernutrition categories for the nine tribes. This analysis notably shows a prevalence of more than twofold risk towards acquiring undernutrition (BMI<18.5 kg/m^2^) in the overall category among young females than the males in the same age group. Such risk in this category is as high as more than 6 times (95% CI 2.9–13.9; p≤0.001) among the Oraon and more than five times among the Bhumij (95% CI 1.6–16.6; p≤0.001) along with 2–3 times among four other tribes, namely Kora, Santals of Odisha, Bathudi and Kukna. While considering the status of older females in the category of overall undernutrition, it was observed that there is a significant risk for older Kora females with an odds of 2.6 (95% CI 1.2–5.5; p≤0.01) in comparison to their male counterparts; in case of other tribes the odds of risk is not significant.

**Table 7:** (A-B). Odds for undernutrition among females against males in <40 year and ≥40 year age categories.

**A**							
**Sl No**	**Names of the tribes**	**Odds undernutrition (<18.5 kg/m2) among females**		**Odds for combined moderate and mild undernutrition (≥16 to <18.5 kg/m2) among females**		**Odds for severe undernutrition (<16 kg/m2) among females**	
		**<40 years**	**≥40 years**	**<40 years**	**≥40 years**	**<40 years**	**≥40 years**
1	Santal (WB)	1.4 (0.6–3.0)*	1.7 (0.8–3.5)	1.3 (0.6–2.1)	1.4 (0.6–2.0)*	3.9 (0.8–19.5)#*	2.6 (0.5–14.2)*
2	Kora	2.1 (1.0–4.4)*	2.6 (1.2–5.5)**	1.2 (0.6–2.5)	1.0 (0.5–2.1)	7.0 (1.5–32.7)**	8.0 (2.2–29.0) ***
3	Oraon	6.3 (2.9–13.9)***	1.6 (0.8–3.4)	3.91 (1.8–8.4)***	0.6 (0.3–1.3)	NA	3.8 (1.4–10.4) **
4	Santal (O)	2.9 (1.3–6.5)**	0.5 (0.2–1.1)	2.6 (1.1–6.0)*	0.4 (0.2–1.0)#*	3.1 (0.3-30-8)	1.0 (0.2–4.3)
5	Bhumij	5.2 (1.6–16.6)***	2.0 (0.9–4.1)#*	4.4 (1.4–14.2)**	2.0 (0.9–4.5)#*	NA	0.75 (0.2–3.5)
6	Bathudi	3.7 (1.8–7.9) **	1.8 (0.9–3.7)	1.6 (0.7–3.3)	0.8 (0.4–1.7)	8.1 (1.8–37.8)**	4.1 (1.4–12.1) **
7	Dhodia	1.6 (0.8–3.5)	0.6 (0.3–1.4)	1.9 (0.9–4.3)	0.4 (0.2–1.1)#*	0.8 (0.2–3.2)	2.0 (0.4–11.6)
8	Kukna	2.5 (1.1–5.48)*	1.2 (0.6–2.5)	2.6 (1.1–5.9)*	1.4 (0.7–3.0)	1.6 (0.3–9.8)	NA
9	Chaudhari	1.2 (0.6–2.6)	1.7 (0.8–3.4)	0.7 (0.3–1.5)	1.0 (0.5–2.0)	4.8 (1.3–17.8) *	3.1 (0.9–10.3)#*
	Total	2.2 (1.1–4.6)*	1.7 (0.8–3.5)	1.9 (0.9–4.0)	1.36 (0.6–3.0)	3.9 (0.8–19.5)#*	2.6 (0.5–14.2)
B							
**Sl No**	**Names of the tribes**	**Odds for moderate undernutrition (≥16 to ≤16.99 kg/m2) among females**		**Odds for mild undernutrition (≥17 to <18.5 kg/m2) among females**			
		**<40 years**	**≥40 years**	**<40 years**		**≥40 years**	
1	Santal (WB)	**3.0 (0.8–11.7)**#*	1.7 (0.5–5.5)	1.3 (0.6–3.1)		1.1 (0.5–2.7)	
2	Kora	1.7 (0.6–5.1)	**2.3 (0.7–6.9)**#*	0.9 (0.4–2.0)		0.6 (0.3–1.4)	
3	Oraon	7.4 (1.6–34.4)**	0.9 (0.3–2.8)	**2.0 (0.9–4.5)**#*		0.5 (0.2–1.4)	
4	Santal (O)	2.1 (0.4–11.8)	0.3 (0.03–3.2)	2.4 (1.0–6.0)*		0.4 (0.2–1.2)	
5	Bhumij	**NA**	1.5 (0.4–5.0)	**3.1 (1.9–10.1)**#*		2.0 (0.8–4.9)	
6	Bathudi	2.2 (0.6–7.7)	1.6 (0.5–5.2)	1.2 (0.6–2.7)		0.6 (0.3–1.4)	
7	Dhodia	8.1 (1.0–7.6)*	0.2 (0.02–1.6)	1.2 (0.5–2.8)		0.6 (0.2–1.9)	
8	Kukna	2.6 (0.6–10.6)*	1.3 (0.5–3.7)	**2.1 (0.9–5.3)**#*		1.3 (0.5–3.2)	
9	Chaudhari	1.0 (0.2–4.2)	0.8 (0.3–2.6)	0.6 (0.3–1.5)		1.1 (0.5–2.4)	
	Total	2.8 (1.7–4.5)***	1.2 (0.8–1.8)	1.4 (1.1–1.9)**		0.9 (0.6–1.1)	

* p is significant at ≤0.05,

** p is significant at ≤0.01,

*** p is significant at ≤0.001,

^#^* p is 0.09≥p≥0.05.

While considering moderate undernutrition, it is observed that there is as high as 3 times (approximately) (95% CI, 1.7–4.5; p≤0.001) risk for the young women to acquire undernutrition in comparison to the young men. Young women belonging to the tribes like Santals of West Bengal, Oraons, Kuknas and Dhodias show very high risk in this category.

In the mild undernutrition category, there is a 1.4 time (95% CI 1.1–1.9; p≤0.01) risk for young females to come under the stress of undernutrition in comparison to males in the same age category. Females from Santals of Odisha, Oraon, Bhumij and Kukna tribes show significantly higher risks towards undernutrition in young age groups.

In the combined mild and moderate undernutrition category, young females exhibited a risk ranging from 2.5 to 4.4 times in 4 of the 9 tribes (Table 7A).

In the overall undernutrition category, the young females in four of the nine tribes (Oraon, Santals of Odisha, Bhumij and Kukna) consistently showed a significantly higher risk towards acquiring undernutrition in comparison to their male counterparts. On the other hand, only Santals of West Bengal showed a significantly higher risk towards acquiring the combined mild and moderate undernutrition in the older age category (Table 7A). The trend further showed that older females in 6 of the 9 tribes are at a lower risk of acquiring undernutrition in comparison to their male counterparts. Our observations showed that the lesser odds towards acquiring combined mild and moderate undernutrition among the older females was not due to the low prevalence of undernutrition than their male counterparts, rather the prevalence rate was similar or very close among the males and females in older age group in maximum number of tribes.

In severe undernutrition category (Table 7A) the tribal females are showing a very high risk towards acquiring undernutrition with statistically significant odds. It is observed that Santals of West Bengal, Kora, Bathudi and Chaudharis are the tribes that are under significantly higher risk of undernutrition in both the age groups. Overall, the young females show an approximately 4 times higher risk than their male counterparts.

## Discussion

In the context of increasing concern in nutritional insecurity of Indian marginalized sections, present study highlights the continuing as well as the increasing risk of high undernutrition among Indian tribes in comparison to the rest of the Indian population. The findings of the present study are important as they clearly highlight a serious state of malnutrition among tribal women exhibiting a grimmer nutritional status than their male counterparts. The analysis further exhibits that nutritional status of the females among Indian tribes is 9, 4, 1.5, 6 and 15 percentage points below the status of males with respect to prevalence of undernutrition in its severe, moderate, mild, combined moderate and mild, and overall categories respectively. These results can be attributed to the intra-society gender discrimination in attending food security among Indian tribes (Figs 5 and 6) along with the marginalization at inter-society level, matching similar findings reported previously [40].

The high prevalence of undernutrition during infancy and childhood plays a defining role in causing undernutrition in the adolescent, youth and the later stages of the life [17]. High undernutrition in the first two years of life of an individual mostly, leads to growth related permanent health problems like stunting or wasting in the following period of life. It also contributes towards chronic energy deficiency (CED) and other micro-nutrient deficiency in the youth and the advanced age [16, 18]. In the highly disadvantaged nutrition scenario among Indian tribes, severe malnourishment carry very high risk for the tribal infants and children by carrying forward the undernutrition status further to the adolescence as well as to later stages in their lives. So, the findings of the present study demonstrating the heavy burden of undernutrition in the adulthood among Indian tribes have possible reasons in the severe stress of infant and childhood undernutrition.

Evidences show that stunting or severe undernutrition is an additional risk factor among socio-economically marginalized groups [41, 42]. National Family and Health Survey-III report [19] shows that about 85% of the tribal households in India belong to the two poorest wealth quartiles. This extreme poverty is a leading cause of high malnutrition among the tribes of India by restricting their buying capacity to a substantially lower level. Various studies among the tribal children in India showed a high prevalence of malnutrition in the form of wasting and stunting [30, 42, 43]. On the other hand, the present study demonstrates a high undernutrition among the tribal women, which explains their probable suffering from stunting and wasting in their childhood that has continued to the adolescence and further to their youth and later stages in their lives. Additionally, most tribal women get married in an early age [19, 44] and attain motherhood;, their malnourishment driven ill-health gets continued to their motherhood. Young women suffering from undernutrition and ridden with other risk factors like early age marriage, poverty, illiteracy are at increased odds for their children towards developing stunting and wasting at a twofold risk [1, 45, 46]. Findings have shown that when an underweight young or adolescent girl becomes a mother, she fails to support her own growth alongside the fetus, which often leads the young mother in giving birth to underweight babies [47–49]. In India, 41% and 53% babies born to moderately and severely underweight mothers respectively were underweight (less than 2,500 g) at the time of birth [50]. So, undernutrition among women and their children when left unaddressed in one generation, may get carried on to the next generations forming a vicious circle which leads to the gross breakdown of the health of the community, leading to high prevalence of the nutritional extreme among them. It is, thus noteworthy that the policy planning for addressing undernutrition needs to consider poverty and other associated factors concomitantly while implementing the nutritional supplement program.

In the context of a highly volatile economic condition, widespread and continuing drought and food scarcity, there has been a steep inflation in food prices in the last few years in India [51]. There are few specific observations or reviews on the food substitution and nutritional management practices being adopted by the underprivileged groups like Indian tribes at their household levels. As per the Guardian report (2011, 2013) most of the world's poor live in middle-income countries (MIC), and India in particular with her improvement of income status to MIC does not bring any large difference for the poor residing within [52, 53]. The Census of India (2011) further indicated that the Indian tribes that constitute 8.6% of the total Indian population experience a severe poverty and high marginalization on their economic front [27]. In the present research, we also tried to explore the status of migration among Scheduled Tribe (ST) communities as it is an important income generating event among many of the backward communities in India. We observed fewer inter-state migration among the studied tribes as well as migration to the distant urban or industrial places within states. Our findings further reveal that exploitation at work places and non-payment of wages are few of the many reasons that prevent them from moving to the other places for income generation. These are the major possible contributing factors of their continuing non-affordability and chronic poverty which might be leading to their increased food insecurity and severe state of malnourishment.

Tribal families are mostly covered under Below Poverty Line (BPL) schemes implemented by different agencies of the state and the central government. They are supplied with rice, wheat and sugar at highly subsidized rates under nutrition supplement programs [54]. On the other hand, their traditional practice of food collection from the forest has got restricted due to various forest protection acts [55, 56]. With the increased inflation rate in food prices, these economically backward groups belonging to the lowest socioeconomic strata of the Indian society are failing to afford diversity in their food basket [45, 57, 58] that they previously maintained. This is possibly another leading cause of their undernutrition in both the forms of micronutrient and energy supplement diet.

NNMB (2009) and India Health Report (2015) showed that there was no gender discrepancy regarding food access among preschool tribal children (<5 years) and adolescents [26, 59]. Similar observations have also been made by NFHS-III [19]. However, UNICEF (2013) suggested a possible gender dimension in childhood as a factor affecting access to food in the tribal society [12]. The present study finds a highly discriminated prevalence (Figs 5 and 6) and risk (Tables 3, 7A and 7B) of undernutrition among the young females than their male counterparts. On the other hand, research has shown that social and cultural issues like early age marriage, high school drop-outs rate etc. which are highly prevalent among tribes are the important causes of nutritional extreme among the females in their youth [45].

It would be worth mentioning here that male members of the studied tribes were observed to have easy access to nutritional and diet facilities as compared to the female members because males receive early and extensive social freedom to access income generating activities. Young women, on the other hand in the tribal societies are mostly confined to the domestic and household work by keeping away from most of the income generating activities which broadly limit their buying capacity and independent access to food by broadly affecting their nutrition. A similar observation was also made in another study [45].

Some important points have emerged out from the present study. In the studied tribes, the extent of mild undernutrition is conspicuously high among the females. Similarly, moderate undernutrition among the females was almost double in comparison the males among six of the nine tribes. Furthermore, the prevalence of severe undernutrition among the females was more than four times in four tribes (Table 2A and 2B) and three times in another one tribe (Table 2A and 2B). Such findings highlight the increase in extent of marginalization in access to food with the increase in nutritional extreme. This is a clear indication of the non-inclusiveness of the females at the community level in general, and their marginalization at individual level in particular.

Furthermore, the higher proportion of older women (≥50 years) suffering from severe undernutrition (Fig 7) indicates their chronic nutritional insecurity status in comparison to younger women (<50 years).

Thus, considering the deep-rooted social disadvantages and marginalization of Indian tribes on various fronts, improvement of their health and nutritional conditions must be clustered with their socioeconomic development with a focus on gender parities.

Observing the high heterogeneity in the prevailing status of undernutrition at inter-tribal level, a tribe-wise approach in addressing such issues will bring a much significant outcome. Considering difficult terrain of habitation, varying life style pattern and most importantly the unique socio-cultural milieu of Indian tribes, it is desirable to prioritize and address such health issues with a focus on marginalized tribal populations for an effective health care delivery system. Empowerment of the community perspectives in nutritional security program by encouraging community level initiatives can bring successful results. Strengthening the nutritional supplementation program for the adolescents in the areas where the program is ineffective can bring better results. Pre-age marriage and early pregnancy are other important causes of high undernutrition among young women. Thus, strengthening of maternal nutrition and health, particularly of young mothers hold the key to arrest the nutritional problem from being inherited. Additionally, studies focusing on various aspects of undernutrition among the older population of Indian tribes need to be undertaken. Explanations in behavioral and socio-cultural aspects involving women that influence their access to food and affect various power dynamics, gender role etc. at household and community level will bring meaningful inputs to improve their nutritional status.

We acknowledge certain limitations in the present study. We do not have data and specific observations on the tribal participants from remote areas, which may further broaden the knowledge on undernutrition landscape and the health status of women as people living in those areas face difficulties in accessing government health facilities as well as other basic amenities. There are few other important aspects, though were not within the scope of the objectives of the present study, could have brought additional insights to the problems of undernutrition among the tribal women. Firstly, we did not collect any behavioral data and therefore we cannot establish any linkages between nutritional status and food consumption and sufficiency. Secondly, our research did not look into the socio-cultural aspects of power dynamics and gender dynamics within the households. Finally, our analysis did not compare the nutritional perspectives of same tribes (Santals) living in different geographical areas. Studies considering such dimensions can bring further meaningful perspectives to the status of tribal nutrition in general and gender based discrimination in undernutrition in particular.
